# Metabolic and Body Composition Risk Factors Associated with Metabolic Syndrome in a Cohort of Women with a High Prevalence of Cardiometabolic Disease

**DOI:** 10.1371/journal.pone.0162247

**Published:** 2016-09-02

**Authors:** Philippe Jean-Luc Gradidge, Shane A. Norris, Nicole G. Jaff, Nigel J. Crowther

**Affiliations:** 1 Centre for Exercise Science and Sports Medicine (CESSM), Faculty of Health Sciences, University of the Witwatersrand, Johannesburg, South Africa; 2 MRC/Wits Developmental Pathways for Health Research Unit, Faculty of Health Sciences, University of the Witwatersrand, Johannesburg, South Africa; 3 Department of Chemical Pathology, National Health Laboratory Service, Faculty of Health Sciences, University of the Witwatersrand, Johannesburg, South Africa; Universita degli Studi di Catania, ITALY

## Abstract

**Background:**

The aetiology of the metabolic syndrome and the inter-relationship between risk factors for this syndrome are poorly understood. The purpose of this investigation was to determine the risk factors for metabolic syndrome and their interactions in a cohort of women with a high prevalence of metabolic syndrome.

**Materials and Methods:**

Abdominal and whole body composition (ultrasound and dual-energy X-ray absorptiometry), blood pressure, and cardiometabolic and demographic factors were measured in a cross-sectional study of 702 black African women from Soweto, Johannesburg. Data was analysed using multivariate logistic regression.

**Results:**

Metabolic syndrome was present in 49.6% of the study cohort. Logistic regression analysis demonstrated that adiponectin (odds ratio [95% CIs]: 0.84 [0.77, 0.92], p<0.0005) and abdominal subcutaneous fat (0.56 [0.39, 0.79], p = 0.001) reduced metabolic syndrome risk whilst insulin resistance (1.31 [1.16, 1.48], p<0.0005) and trunk fat-free soft-tissue mass (1.34 [1.10, 1.61], p = 0.002) increased risk. Within this group of risk factors, the relationship of adiponectin with metabolic syndrome risk, when analysed across adiponectin hexiles, was the least affected by adjustment for the other risk factors.

**Conclusions:**

Adiponectin has a significant protective role against metabolic syndrome and is independent of other risk factors. The protective and possible augmentive effects of abdominal subcutaneous fat and lean trunk mass, respectively on metabolic syndrome risk demonstrate the existence of novel interactions between body composition and cardiometabolic disease.

## Background

Metabolic syndrome (MetS), which is one of the major pandemics affecting health across the globe [1], is characterised by a clustering of cardiometabolic factors which increase the risk of cardiovascular disease and type 2 diabetes [2]. A complete understanding of the aetiology of the MetS is difficult to achieve due to the presence of multiple components, each of which have their own individual pathophysiological origins. Furthermore, the MetS includes metabolic (lipids and glucose), cardiovascular (blood pressure) and anthropometric (waist circumference) factors with the latter of these itself having an aetiological input into each of the other components [3]. Thus, when investigating the aetiology of the syndrome, risk factors may be revealed simply because of their correlation with waist circumference.

A number of risk factors for MetS have been demonstrated [4] however, there is little data on how disease risk varies across the range of levels of these variables or whether individual factors modulate the disease risk contributed by other identified risk markers. Such information would be important to our understanding of the aetiology of the MetS and may improve our ability to identify true markers of disease risk. The identification of risk factors for MetS and a greater understanding of their inter-relationships may be accomplished by the investigation of populations with high levels of cardiometabolic disease prevalence. This is true for urban South African females in whom the prevalence of both obesity and MetS are high [5] and little is known about the disease aetiology.

Therefore, the aims of the current study were as follows: to identify the main contributing factors to the cardiometabolic features of the MetS in a cohort of urban African women known to have a high prevalence of MetS; to determine how disease risk varied across the range of levels of each risk factor; to examine whether each risk factor modulated the contribution of the other factors to disease risk across their range and to determine which individual components of the MetS were influenced by each of the risk factors. We hypothesised that each risk factor would modify the contribution of the other factors to disease risk by varying degrees across their range. We further hypothesised that each of these factors will influence MetS risk by effects on individual components of the syndrome. The risk factors that we chose to study were the more ‘classical’ risk factors including markers of body composition and insulin sensitivity and environmental factors including employment and education status and tobacco use, and factors that were more specific to the study population such as menopausal and HIV status. It was hoped that this investigation would identify the principal risk factors for MetS and reveal novel information on the interplay between these factors in modulating disease risk.

## Materials and Methods

### Study Population

This cross-sectional study of African women comprised biological mothers and/or caregivers of children from the Birth-to-Twenty (Bt20) study [6]. The Bt20 longitudinal study began in 1990 with the enrolment of 3 273 pregnant women in the greater Johannesburg metropolitan region. The population is representative of the demographic characteristics of long-term residents of Soweto, Johannesburg [7]. A total of 2 200 caregivers/mothers remain in communication with the study administrators, of whom 867 were eligible for recruitment into the current study based on the following criteria: age between 40–60 years, not being pregnant and being black South African. However, 165 of this group could not participate in the study, for the following reasons: refusal (n = 79), uncontactable (n = 46), died (n = 37), and terminally ill (n = 3). Thus, 702 women volunteered to participate, and underwent an informed consent process. Ethical clearance was granted by the Human Research Ethics Committee (Medical), University of the Witwatersrand, and written consent obtained from all participants.

### Anthropometric Measurements

The protocols for dual-energy X-ray absorptiometry (DXA) body composition measurements (total body, central (trunk) and peripheral (arms and legs) fat mass, and total body, central and peripheral fat-free, soft-tissue mass (FFSTM)), and simple body composition measurements (waist and hip circumference, BMI) have been reported previously [8, 9]. In this study, the GE LOGIQ ultrasound system (USS) (GE Healthcare, Piscataway, NJ) was used to determine the thickness of visceral (VAT) and subcutaneous adipose tissue (SCAT) with a 2 to 5 MHz 3C-RS curved array transducer located one centimetre above the umbilicus. The ultrasonographic measurements were defined as the distances (depth set at fifteen centimetres) from the peritoneum to vertebrae (for VAT), and the depth (distance set at nine centimetres) from the surface of the skin to the linea alba (for SCAT). A single trained sonographer administered all the measurements (CV<2%, calculated on repeated duplicate measurements on fifteen subjects).The methods used for SCAT and VAT measurement by ultrasonography have been previously validated [10].

### Biochemistry

The protocol for the collection of fasting blood samples, subsequent aliquoting, and storage of serum and plasma samples have been detailed previously [11]. The ADVIA Centaur XP Immunoassay System (Siemens Diagnostics, Tarrytown, USA) was used to perform immunoassays for fasting insulin, and the total CV range for the fasting insulin immunoassay was 4.8 to 5.9%. Insulin resistance was calculated using the HOMA method [12]. The ADVIA 1800 Chemistry System (Siemens Diagnostics, Tarrytown, USA) was used to measure lipid levels (total cholesterol [CV, 0.3%]; HDL [CV, 1.0 to 2.3%]; and serum triglycerides [CV, 0.5 to 1.6%]) and indicators of diabetes mellitus (fasting glucose [CV, 0.4 to 0.5%] and glycated haemoglobin (HbA_1c_) [CV, 0.8 to 1.3%]). The LDL was estimated using the Friedewald formula [13]. Leptin was measured using an ELISA kit (Biovendor Research and Diagnostic Products, Candler, NC) with a CV range of 4.2 to 7.6%. Total adiponectin was quantified using an ELISA assay kit (R&D Systems Inc., Boston Biochem, Cambridge, MA) with a CV range of 2.5 to 4.7%.

### Diagnosis of Disease and Assessment of Menopausal Status

The Omron M6 (version HEM-7001-E, Omron, Kyoto, Japan) was used to record brachial blood pressure (BP). Three measurements were taken with the participant in the seated position with the cuff around the right upper arm, supported at the level of the heart. The average of the last two BP measurements was recorded. The cut-points used for diagnosis of each of the individual components of the MetS were those from the harmonized guidelines [2]. The MetS was diagnosed using 2 different methods: waist circumference was excluded from the assessment and the syndrome was diagnosed when 3 or more of the remaining 4 criteria were met; waist circumference was included and diagnosis was based on the presence of 3 or more of the 5 criteria as prescribed by the harmonized guidelines [2]. The HIV and menopausal status of subjects was assessed as described previously [14].

### Socioeconomic Status

Proxy measures of socioeconomic status, namely, highest level of education and employment status, and tobacco and smokeless tobacco (snuff) use were determined using a validated general health questionnaire [15].

### Statistical Analyses

All analyses were performed using Statistica (version 12, StatSoft, Tulsa, USA). Continuous, normally distributed variables are presented in tables and text as mean ± SD whilst continuous variables with a skewed distribution are shown as median (interquartile range). The latter variables were log transformed to normality before being analysed. Metabolic and anthropometric differences were compared between women with and without MetS (diagnosed either with or without the waist criterion) using either a Student’s unpaired t-test for continuous data or a χ^2^ test for categorical data.

Univariate followed by multivariate logistic regression analyses were performed to identify risk factors for MetS as defined by the criteria that includes waist, and in a separate but identical process risk factors were identified for MetS defined using criteria that excludes waist. The initial univariate logistic regression models included each variable from a list of scientifically plausible variables with MetS as the outcome variable. These variables were: education (completed or did not complete high school), age, employment status (employed or unemployed), snuff use (“yes” or “no”), smoking (“never” as reference versus “current” and “former”), menopausal status (pre- or postmenopausal), HIV status (HIV-negative as reference versus HIV-positive, antiretroviral therapy (ART)-naïve and HIV-positive, receiving ART), adiponectin, leptin, HOMA, subcutaneous and visceral adipose thickness, total body fat, total FFSTM, hip and waist circumferences. The variables that were associated with MetS risk in the univariate logistic regression models with p<0.10 were all included in a single multivariable logistic regression model with MetS again as the outcome variable. Backward, stepwise removal of non-significant variables was performed until only variables with a significant odds ratio (OR) remained in the final model. Collinearity within this model was quantified via variance inflation factor (VIF) analysis, but none was observed with all VIFs<5.00. Each of the independent continuous variables that remained in the multivariable logistic regression model for MetS diagnosed without the criterion for waist were then analysed as hexiles, with the lowest hexile used as the reference, in the presence and absence of each of the other independent variables that significantly associated with MetS. This allowed us to determine how risk for MetS varied across the range of each independent variable and whether this was affected by the other significant modifiers of MetS risk. In addition, the risk factors for each component of the MetS (except waist) were determined using multivariable, backward step-wise logistic regression analysis as described above.

## Results

### Subject Characteristics

Body composition and metabolic features of the participants with or without MetS are presented in Table 1. MetS was diagnosed using only HDL, triglyceride, glucose and blood pressure measurements, and 3 or more of these 4 measurements had to exceed the cut points defined by the harmonised guidelines [2]. The prevalence of MetS using these criteria was 14.0%, whilst the prevalence using the full set of five criteria [2], was 49.6%. The prevalence of the individual components of the MetS were as follows: waist circumference ≥80 cm, 90.5%; hypertension, 64.7%; fasting glucose ≥5.6 mmol/l, 16.3%; triglyceride ≥1.7 mmol/l, 15.4%; HDL <1.3 mmol/l, 66.2%. The prevalence of obesity was 67.8%, of extreme obesity (BMI ≥ 40) was 16.8%, of LDL ≥3 mmol/l was 36.4% and of total cholesterol ≥5 mmol/l was 29.9%. The prevalence of diabetes was 8.9% of whom 77.2% were receiving therapy. Within the total cohort of 702 subjects, 27.4% were receiving therapy for hypertension, whilst 2.28% were receiving therapy for dyslipidaemia. The prevalence of HIV infection (21.3%) and ART use (55.3% of HIV-infected subjects were receiving ART) has been reported previously [14]. The data in Table 1 demonstrates that nearly all the anthropometric and cardiometabolic variables were higher in the women with MetS than those without, whereas socioeconomic and education status were similar between the two groups. When MetS was diagnosed using the full set of 5 criteria from the harmonised guidelines [2] (S1 Table), it was observed that in women with MetS both leg fat (14.8 ± 4.31 vs. 13.5 ± 4.72 kg; p<0.0005) and leptin (31.1 [18.6, 45.9] vs. 22.7 [13.1, 40.5]; p<0.0005) were significantly higher than in subjects without MetS. However, such differences were not observed for the women depicted in Table 1. In addition, subcutaneous fat thickness was not different (p = 0.083) between women with (3.49 ± 1.01 cm) or without (3.35 ± 1.03 cm) the MetS when diagnosed using the full criteria, but when using the criteria without waist, subcutaneous fat thickness was lower in women with MetS (p<0.05; see Table 1).

**Table 1:** Anthropometric and metabolic variables in women with and without metabolic syndrome^a^.

Variables	Women with metabolic syndrome^a^ (n = 90)	Women without metabolic syndrome (n = 552)	P-value
Age (years)	51.0 ± 5.21	49.0 ± 5.19	0.001
BMI (kg.m^-2^)	35.4 ± 6.75	32.8 ± 7.26	0.002
Waist (cm)	106 ± 11.2	97.5 ± 14.4	<0.0005
Hip (cm)	121 ± 13.3	117 ± 14.8	0.02
Arm fat (kg)	4.13 ± 1.08	3.63 ± 1.26	0.001
Arm fat-free, soft-tissue mass (kg)	4.86 ± 0.89	4.36 ± 0.80	<0.0005
Leg fat (kg)	14.0 ± 4.22	14.1 ± 4.64	0.80
Leg fat-free, soft-tissue mass (kg)	16.5 ± 2.81	15.4 ± 2.82	0.001
Trunk fat (kg)	16.5 ± 4.15	14.0 ± 5.13	<0.0005
Trunk fat-free, soft-tissue mass (kg)	23.7 ± 3.26	21.4 ± 3.18	<0.0005
Total body fat (kg)	34.6 ± 8.47	31.8 ± 10.2	0.02
Total fat-free, soft-tissue mass (kg)	45.0 ± 6.57	41.2 ± 6.55	<0.0005
Subcutaneous fat thickness (cm)	3.21 ± 0.74	3.45 ± 1.06	0.04
Visceral fat thickness (cm)	5.07 ± 1.56	4.29 ± 1.72	<0.0005
Systolic blood pressure (mmHg)	143 [130, 159]	128 [117, 143]	<0.0005
Diastolic blood pressure (mmHg)	93.0 [87.5, 101]	86.0 [77.5, 94.5]	<0.0005
HbA_1c_ (%)	6.30 [5.90, 7.70]	5.80 [5.50, 6.20]	<0.0005
Fasting glucose (mmol/l)	5.70 [5.00, 7.20]	4.70 [4.40, 5.10]	<0.0005
Insulin (pmol/l)	13.5 [8.60, 20.0]	9.80 [6.40, 14.1]	<0.0005
HOMA	3.93 [2.22, 5.69]	2.01 [1.31, 3.10]	<0.0005
Adiponectin (μg/ml)	4.54 [3.33, 6.77]	7.52 [4.94, 11.2]	<0.0005
Leptin (ng/ml)	28.0 [18.7, 45.3]	27.3 [14.8, 43.6]	0.56
Total cholesterol (mmol/l)	4.48 ± 1.12	4.49 ± 1.05	0.94
LDL (mmol/l)	2.66 ± 0.99	2.73 ± 0.88	0.47
HDL (mmol/l)	1.00 [0.90, 1.10]	1.20 [1.00, 1.50]	<0.0005
Triglycerides (mmol/l)	1.80 [1.20, 2.10]	1.00 [0.80, 1.30]	<0.0005
Employed (%)	53.3 (42.8, 63.8)	57.5 (53.4, 61.7)	<0.0005
Completed high school (%)	33.3 (23.2, 43.4)	29.6 (25.7, 33.4)	<0.0005
Smokers (%)	13.3 (6.17, 20.5)	7.43 (5.23, 9.62)	<0.0005
Consume snuff (%)	13.5 (6.25, 20.7)	21.8 (18.4, 25.3)	<0.0005

^**a**^Metabolic syndrome diagnosis was made in subjects in whom 3 or more of the following 4 variables exceeded the cut points set out by the harmonised guidelines [2]: blood pressure, glucose, triglyceride and HDL levels; data expressed as mean ± SD, median [interquartile range] or % (95% CIs)

### Univariate and Multivariate Logistic Regression Analyses of Metabolic Syndrome Risk

In separate, univariate, logistic regression models the following variables were associated with MetS (diagnosed using only HDL, triglyceride, glucose and blood pressure measurements) risk at p<0.10: HOMA, adiponectin, waist and hip circumference, total body fat mass, total body FFSTM, subcutaneous and visceral fat thickness, age, menopausal status, receiving ART and smoking. These variables were then included in the same multivariable logistic regression model with MetS as the outcome variable. Following backward, stepwise removal of variables with no significant (p>0.05) association with MetS, the following variables remained in the final model: HOMA, adiponectin, total body FFSTM, subcutaneous fat thickness, age and smoking. Total body FFSTM is a composite variable of leg, arm and trunk FFSTM and therefore these variables were included in the initial multivariate logistic regression model (without total body FFSTM) and the backward stepwise removal of non-significant variables was repeated resulting in the final logistic regression model shown in Table 2. It can be seen that trunk FFSTM remained in the model alongside the same variables described above i.e. HOMA, adiponectin, subcutaneous fat thickness, age and smoking. When a multivariable logistic regression model was built for MetS diagnosed using the full criteria, the final model contained HOMA, adiponectin, age and trunk FFSTM but not subcutaneous fat thickness or smoking (see Table 2).

**Table 2:** Logistic regression models showing significant risk factors for metabolic syndrome diagnosed using criteria with or without inclusion of waist circumference.

Model number	Categorical variable	Independent variables	Odds ratio (95% CI’s);p-value
***1***	Presence of metabolic syndrome (criteria without waist)	Trunk fat-free, soft-tissue mass	1.34 (1.10, 1.61); 0.002
		Subcutaneous fat	0.56 (0.39, 0.79); 0.001
		Adiponectin	0.84 (0.77, 0.92); < 0.0005
		HOMA	1.31 (1.16, 1.48); < 0.0005
		Age	1.10 (1.04, 1.16); 0.001
		Smoking	3.07 (1.28, 7.33); 0.01
***2***	Presence of metabolic syndrome (criteria with waist)	Trunk fat-free, soft-tissue mass	1.19 (1.11, 1.27); <0.0005
		Adiponectin	0.94 (0.91, 0.98); 0.004
		HOMA	1.31 (1.16, 1.47); <0.0005
		Age	1.08 (1.04, 1.12); <0.0005

### Risk Factors for Each of the Individual Components of the Metabolic Syndrome

Table 3 displays backward, stepwise multivariable logistic regression models for each of the 4 cardiometabolic components of the MetS. Visceral fat, HOMA, age, and smoking are associated with an increased risk of high serum triglyceride levels, whilst adiponectin and leg fat are associated with reduced risk (model 1). In model 2, snuff use and adiponectin protect against low HDL levels, whereas trunk FFSTM is associated with a higher risk of low HDL levels. Model 3 shows that adiponectin and subcutaneous fat reduce the risk of impaired fasting glucose, whereas age and HOMA increase the risk. Age and waist circumference are each associated with an increased risk of hypertension (model 4). Neither menopausal or HIV status was associated with any of the individual components of the MetS.

**Table 3:** Logistic regression model showing significant risk factors for the individual components of the metabolic syndrome.

Model number	Categorical variable	Independent variables	Odds ratio (95% CI’s); p-value
***1***	***Triglyceride ≥ 1*.*70 mmol/l***	Visceral fat	1.22 (1.04, 1.44); 0.02
		Leg fat	0.85 (0.79, 0.92); < 0.0005
		Adiponectin	0.92 (0.86, 0.98); 0.006
		HOMA	1.11 (1.00, 1.24); 0.05
		Age	1.07 (1.02, 1.12); 0.007
		Smoking	2.53 (1.21, 5.30); 0.01
***2***	***HDL < 1*.*30 mmol/l***	Trunk fat-free, soft-tissue mass	1.14 (1.04, 1.24): 0.005
		Adiponectin	0.93 (0.90, 0.97); < 0.0005
		Snuff use	0.63 (0.40, 0.99); 0.05
***3***	***Glucose ≥ 5*.*60 mmol/l***	Subcutaneous fat	0.59 (0.42, 0.83); 0.002
		Adiponectin	0.93 (0.87, 1.00); 0.04
		HOMA	1.73 (1.49, 2.00); <0.0005
		Age	1.09 (1.03, 1.15); 0.002
***4***	***Blood pressure ≥ 130/85 mmHg***	Waist	1.05 (1.01, 1.08); 0.005
		Age	1.05 (1.00, 1.11); 0.04

### Risk for Metabolic Syndrome across Hexiles of Each Risk Factor

**Fig 1:**
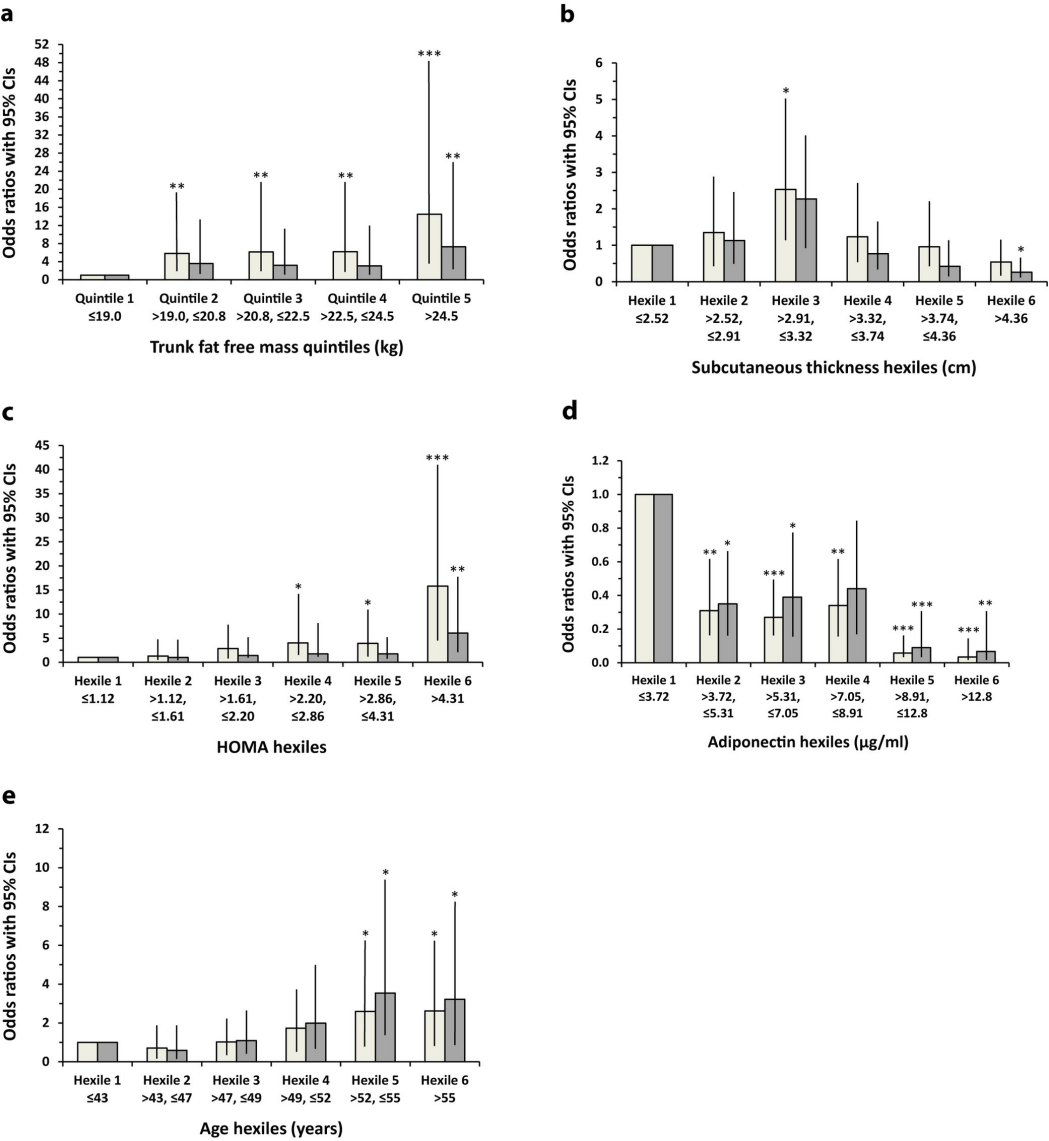
Risk of metabolic syndrome across hexiles/quintiles of: A. trunk fat-free, soft-tissue mass, B. abdominal subcutaneous fat thickness, C. HOMA, D. adiponectin and E. age. Lighter bars represent unadjusted odds ratios whilst darker bars represent odds ratios with adjustment for smoking and all 4 of the other variables shown in this figure; *p<0.05, **p<0.005, ***p<0.0005 vs hexile 1.

Fig 1 shows the odds ratios (ORs) for MetS risk across hexiles (quintiles for trunk FFSTM) of each of the continuous independent variables found in the final multivariable logistic regression model shown in Table 2. The ORs are shown both unadjusted and adjusted for all the other independent variables in the final model (Table 2). Trunk FFSTM was analysed as quintiles because with hexiles only 1 MetS case was observed in the first hexile. The unadjusted ORs for MetS risk are significant for each trunk FFSTM quintile, rising to a maximum in quintile 5 (see Fig 1A). However, after adjustment for all the other variables, all ORs fell with only quintile 5 demonstrating a significant OR (p<0.005). This attenuation of risk was analysed in more detail by adding each of the variables to the model one at a time. It was observed that only the addition of adiponectin to the model caused any of the trunk FFSTM quintiles i.e. quintiles 3 and 4, to become non-significant, suggesting that adiponectin was largely responsible for the risk attenuation. Fig 1B shows a similar analysis of MetS risk across hexiles of subcutaneous fat thickness. The unadjusted ORs for MetS rise from hexile 1 to a peak at hexile 3 and then fall progressively to a nadir at hexile 6. Only hexile 3 shows a statistically significant OR (p<0.05). After adjustment for all the variables, the ORs were all lower, with hexile 3 becoming non-significant but hexile 6 now showing a significantly lower (p<0.05) OR relative to hexile 1. Adding each of the variables on their own to the model or in combination demonstrated that the addition of trunk FFSTM with adiponectin produced a model that mimicked that observed when all variables were added together. The analysis of HOMA hexiles (Fig 1C) shows that for the unadjusted ORs, risk for MetS increased from hexile 1 to reach a maximum at hexile 6, with hexiles 4 (p<0.05), 5 (p<0.05) and 6 (p<0.0005) all showing statistically significant ORs. Adjusting for all the variables reduced all the ORs, leaving only hexile 6 with a significant OR (p<0.005). Further analysis demonstrated that adiponectin in combination with trunk FFSTM were the main contributors to this effect. The data in Fig 1D shows that for adiponectin hexiles, the unadjusted ORs for MetS drop from hexile 1 to 2, plateau till hexile 4 and then drop to hexile 5 with a nadir at hexile 6. All ORs are significantly different to that for hexile 1. After adjustment for all variables the ORs increase slightly but only the OR for hexile 4 becomes non-significant (p = 0.054). When age is divided into hexiles, the unadjusted ORs for MetS rise steadily from hexile 1 to hexile 6, with significant (p<0.05) ORs observed at hexiles 5 and 6 (Fig 1E). A very similar pattern is observed for the fully adjusted ORs, which show no attenuation. With regards smoking, the OR for MetS remained significant with or without adjustment for all the other variables.

## Discussion

This study has shown that in a population of urban African females with a high prevalence of MetS and obesity, trunk FFSTM and abdominal subcutaneous adipose tissue increases and reduces the risk, respectively of MetS. Further analysis demonstrated that this was due to a positive association of trunk FFSTM with risk of low HDL levels and a negative association of subcutaneous adiposity with risk of impaired fasting glucose. Other risk factors for MetS were insulin resistance, age and smoking whilst adiponectin, at all levels across its range, was associated with lower risk. The risk of MetS associated with the other variables, specifically at low levels and with the exception of age, was attenuated after adjusting for adiponectin.

Metabolic syndrome was defined in this study without the waist circumference criteria. This was done because the main aim of our study was to isolate risk factors for MetS that were modulators of the cardiometabolic rather than the anthropometric components of the syndrome. There are many CVD risk factors that correlate with waist circumference and therefore it is reasonable to suggest that a number of these factors may associate with MetS simply through their relationship with waist circumference. It is also possible that the inclusion of waist circumference within the criteria for MetS masks the true relationship of other factors with the cardiometabolic components of the syndrome, as exemplified in the current study for subcutaneous abdominal fat. The definition of MetS in our study required the presence of 3 out of 4 of the cardiometabolic components. This ensured that all subjects with the syndrome as defined by our criteria would also have been included in the cohort of subjects defined using the normal harmonised criteria [2] and would have a severe cardiometabolic disease risk profile.

The current study is the first to show that subcutaneous abdominal fat attenuates the risk for MetS. The reason that no previous studies have observed this relationship may be due to the fact that it is masked by the inclusion of waist circumference within the criteria for diagnosing MetS. Thus, subcutaneous abdominal fat is only revealed as protective for MetS when waist is removed from the MetS criteria. Furthermore, our data show that high subcutaneous fat level is related to a lower risk of MetS, via the effect of abdominal subcutaneous fat on glucose levels (see Table 3). This could possibly be explained by subcutaneous fat acting as a triglyceride reservoir, thus reducing its deposition in visceral fat or at ectopic sites such as muscle, liver or pancreas where lipid deposition leads to increased insulin resistance [16]. Furthermore, a recent large, multinational, cross-sectional study has shown that abdominal subcutaneous fat is associated with a lower risk of type 2 diabetes in women [17], and in a study of 73 type 2 diabetic patients, increased superficial subcutaneous abdominal fat was associated with lower HbA_1c_ and fasting glucose levels [18]. The present study extends the findings of these earlier investigations, which did not measure subcutaneous fat outside of the abdominal area, and demonstrates that it is specifically the abdominal subcutaneous fat depot that is related to improved glycaemia.

Data from the current study demonstrating that age, smoking, insulin resistance and adiponectin are associated with MetS risk are expected, as other studies show similar associations (see Table 2) [4, 19–21]. However, the finding that trunk FFSTM was positively associated with both MetS and low HDL levels, was not expected. Interpretation of this data is complicated by the fact that the DXA-derived trunk FFSTM measure is a composite of all the soft tissue components of the trunk including muscle and organ mass. It is therefore difficult to determine which of these components is the true causative variable. We hypothesise that ectopic fat deposition within the trunk, which DXA is unable to measure, may be the principle element causing these associations. One of the major components of the trunk region is the liver, and it is known that steatohepatitis is characterised by low HDL levels [22]. Future analyses involving a more discriminatory body scanning technique, such as magnetic resonance imaging (MRI), must be undertaken to test this hypothesis. It is interesting to note that an increased risk of MetS with higher whole body FFSTM was observed in a previous study [23], as was an inverse association between HDL levels and non-adipose body mass in both a cross-sectional [24] and a longitudinal study [25]. Furthermore, a recent study has shown that ectopic fat deposition in the liver and skeletal muscle has a greater effect on insulin sensitivity in African than European females [26].

Adiponectin appears in 3 of the 4 logistic regression models describing the risk factors for the 4 cardiometabolic components of the MetS (see Table 3). This demonstrates the wide effect of this adipokine on components of the MetS. Little data are available on the role of adiponectin in the aetiology of MetS in developing countries [27], but studies of African populations do suggest a protective role of adiponectin against MetS [28, 29]. Our data also shows that leg fat protects against elevated triglyceride levels. Such an association has been observed in other studies [30, 31] and may be explained by the hypothesis that subcutaneous fat acts to buffer post-prandial triglyceride and free fatty acid levels [16]. An interesting finding is the positive effect of smokeless tobacco use (snuff) on HDL levels. One other study performed on traditional smokeless tobacco (snus) use in Sweden, also showed a positive association with HDL levels [32]. The mechanism of this association is unknown however, it must be noted that smokeless tobacco formulations differ widely and other studies demonstrate more atherogenic lipid profiles in smokeless tobacco users [33].

Our data shows that for HOMA and trunk FFSTM there is a gradual increase in MetS risk across their range (see Fig 1) and this trend is attenuated after adjustment for all the other independent variables present in the multivariable logistic regression model for MetS (see Table 2), leaving significant ORs only for the highest hexile/quintile. However, it is adiponectin that is the main cause of the attenuation of risk associated with FFSTM, whilst adiponectin and FFSTM explain the attenuation of MetS risk associated with HOMA. This suggests that at lower levels of HOMA and FFSTM adiponectin is a confounder and explains a large proportion of the risk for MetS. Furthermore, risk of MetS drops dramatically with increasing levels of adiponectin and this effect is marginally attenuated by adjustment for the other variables. These results demonstrate that adiponectin across its full concentration range has significant effect on MetS risk and acts independently of the other MetS risk factors, whilst HOMA and trunk FFSTM only have independent effects at high levels. Age also has a significant effect on MetS risk only at the top end of its range, and this effect is not attenuated by adjusting for the other variables. The risk of MetS produced by smoking was also not affected by adjustment for the other variables. With increasing subcutaneous abdominal fat, the OR for MetS rose to a peak by hexile 3 and then fell dramatically by hexile 6. This pattern may be due to confounding because adjustment for the other variables from the logistic regression model caused the peak OR at hexile 3 to fall and significance to disappear, whilst at hexile 6 a significant protective effect appeared. Adiponectin and trunk FFSTM were the main contributors to this effect. This emphasises the influence of adiponectin on MetS risk at lower levels of abdominal subcutaneous fat but an independent protective effect of subcutaneous fat at high levels. These results also demonstrate the strong effect of trunk FFSTM on MetS risk, which may be mediated by ectopic fat, as described previously [22].

This study demonstrates that for most variables the risk they cause for MetS is only observed at the high end of their range, with the risk induced at lower levels mostly due to confounding by other risk factors, most particularly adiponectin. Adiponectin is the only variable that produces significant disease risk across its full range with minimal confounding from other variables. Furthermore, adiponectin modifies MetS risk by effects on multiple components of the syndrome i.e. HDL, triglycerides and glucose tolerance. Hypertension is the only MetS component that is not modulated by adiponectin or HOMA. This suggests that the aetiology of MetS is two-pronged, with insulin resistance-adiponectin being primary aetiological factors for the lipid and glucose sections of the syndrome, whilst age and waist underpin the development of hypertension.

When MetS was defined using the harmonised guidelines [2], 320 such subjects were identified whereas only 90 subjects were identified when MetS was diagnosed without inclusion of the waist criteria. Theoretically, the much lower number of cases identified using the waist-free MetS criteria could lead to a fall in the power of the statistical tests used for comparing cases with controls, which in turn may explain some of the outcomes of our study. However, this was not the case since one of the major outcomes of this study was the identification of new risk factors for MetS when using the diagnostic criteria that did not include waist, rather than the loss of risk factors.

Limitations of this study include its cross-sectional format. Also, abdominal fat levels were not assessed using the gold standard techniques of CT or MRI scanning but rather used ultrasound. However, this method has been favourably validated against MRI [34, 35]. A further limitation of this study was the lack of assessment of ectopic fat deposition, particularly within the liver. There are number of biomarkers that have been associated with an increased risk of MetS including those that indicate oxidative stress [36], inflammation and coagulation [37]. These were not studied in the current investigation because of a lack of data on their association with MetS risk in this population and the emphasis of this study on more classical MetS risk factors. Alcohol consumption was also not measured in this study, and therefore we could not control for it in the analyses. However, this study does have a number of positive attributes including a large sample size and the measurement of a wide variety of appropriate variables in a population for whom little data exists on the aetiology of MetS. This is the only study to determine how risk for MetS varies across the range of each risk factor and how these factors modulate the effect of one another across their ranges. Defining MetS without the use of the waist criteria also allowed us to uncover a novel association with subcutaneous abdominal fat that provides valuable new information on the effects of different body tissue compartments on MetS risk. This study also highlights the effect of trunk FFSTM on the aetiology of cardiometabolic disease.

## Conclusions

In conclusion, this study demonstrates that the cardiometabolic components of the MetS are favourably modulated by various subcutaneous body fat depots and that trunk FFSTM may have detrimental effects on HDL levels via an unknown mechanism that warrants further investigation. Falling adiponectin levels independently affect multiple components of the MetS, significantly reducing MetS risk even at the lower end of its range. Other risk factors have prominent effects on MetS risk only at the upper end of their range and the effects observed at lower levels are largely due to confounding from adiponectin.

## Supporting Information

S1 Table Anthropometric and metabolic variables in women with and without metabolic syndrome. (DOCX)
