# Correction: Metabolic and Body Composition Risk Factors Associated with Metabolic Syndrome in a Cohort of Women with a High Prevalence of Cardiometabolic Disease

**DOI:** 10.1371/journal.pone.0165215

**Published:** 2016-10-17

**Authors:** The PLOS ONE Staff

The first author’s name is incorrect in the citation of the article. The publisher apologizes for the error. The correct citation should be as follows: Gradidge PJ, Norris SA, Jaff NG, Crowther NJ (2016) Metabolic and Body Composition Risk Factors Associated with Metabolic Syndrome in a Cohort of Women with a High Prevalence of Cardiometabolic Disease. PLoS ONE 11(9): e0162247. doi:10.1371/journal.pone.0162247
