# Supplementary material for: Metabolic and Body Composition Risk Factors Associated with Metabolic Syndrome in a Cohort of Women with a High Prevalence of Cardiometabolic Disease
Source: PLoS One. 2016 Sep 2;11(9):e0162247. doi: 10.1371/journal.pone.0162247 (PMC5010252; doi:10.1371/journal.pone.0162247)
Supplement: S1 Table — (DOCX) [file pone.0162247.s001.docx]

**S1 Table. Anthropometric and metabolic variables in women with and without metabolic syndrome^a^**

| **Variables** | **Women with metabolic syndrome^a^ (n=320)** | **Women without metabolic syndrome (n=325)** | **P-value** |
| --- | --- | --- | --- |
| Age (years) | 50.1 ± 5.33 | 48.5 ± 5.13 | <0.0005 |
| BMI (kg.m^-2^) | 35.4 ± 6.6 | 30.9 ± 6.98 | <0.0005 |
| Waist (cm) | 104 ± 11.6 | 93.4 ± 14.4 | <0.0005 |
| Hip (cm) | 122 ± 13.5 | 114 ± 14.6 | <0.0005 |
| Arm fat (kg) | 4.08 ± 1.14 | 3.36 ± 1.25 | <0.0005 |
| Arm fat-free, soft-tissue mass (kg) | 4.70 ± 0.86 | 4.20 ± 0.75 | <0.0005 |
| Leg fat (kg) | 14.8 ± 4.31 | 13.5 ± 4.72 | 0.0005 |
| Leg fat-free, soft-tissue mass (kg) | 16.3 ± 2.77 | 14.9 ± 2.78 | <0.0005 |
| Trunk fat (kg) | 16.1 ± 4.37 | 12.7 ± 5.15 | <0.0005 |
| Trunk fat-free, soft-tissue mass (kg) | 23.0 ± 3.18 | 20.6 ± 3.08 | <0.0005 |
| Total body fat (kg) | 35.0 ± 8.76 | 29.5 ± 10.3 | <0.0005 |
| Total fat-free, soft-tissue mass (kg) | 44.0 ± 6.43 | 39.7 ± 6.26 | <0.0005 |
| Subcutaneous fat thickness (cm) | 3.49 ± 1.01 | 3.35 ± 1.03 | 0.09 |
| Visceral fat thickness (cm) | 4.89 ± 1.54 | 3.94 ± 1.76 | <0.0005 |
| Systolic blood pressure (mmHg) | 138 [128, 150] | 121 [113, 136] | <0.0005 |
| Diastolic blood pressure (mmHg) | 91.5 [86.5, 99.5] | 81.2 [75.0, 90.5] | <0.0005 |
| HbA_1c_ (%) | 5.90 [5.60, 6.50] | 5.70 [5.40, 6.10] | <0.0005 |
| Fasting glucose (mmol/l) | 5.00 [4.60, 5.70] | 4.60 [4.30, 4.90] | <0.0005 |
| Insulin (pmol/l) | 12.0 [7.80, 17.3] | 8.32 [5.65, 12.8] | <0.0005 |
| HOMA | 2.65 [1.75, 4.45] | 1.76 [1.19, 2.69] | <0.0005 |
| Adiponectin (μg/ml) | 5.91 [3.78, 8.62] | 8.47 [5.51, 12.6] | <0.0005 |
| Leptin (ng/l) | 31.1 [18.6, 45.9] | 22.7 [13.1, 40.5] | 0.01 |
| Total cholesterol (mmol/l) | 4.38 ± 1.04 | 4.61 ± 1.05 | 0.55 |
| LDL (mmol/l) | 2.71 ± 0.88 | 2.75 ± 0.90 | <0.0005 |
| HDL (mmol/l) | 1.10 [0.90, 1.20] | 1.40 [1.10, 1.60] | <0.0005 |
| Triglycerides (mmol/l) | 1.30 [0.90, 1.70] | 1.00 [0.70, 1.30] | <0.0005 |
| Employed (%) | 57.5 (52.1, 62.9) | 57.7 (52.3, 63.1) | 0.01 |
| Completed high school education (%) | 26.6 (21.7, 31.5) | 33.6 (28.4, 38.9) | <0.0005 |
| Smokers (%) | 9.37 (6.16, 12.6) | 7.38 (4.53, 10.2) | <0.0005 |
| Consume snuff (%) | 19.1 (14.8, 23.5) | 21.4 (16.9, 25.9) | <0.0005 |

**^a^**Metabolic syndrome diagnosis was made in subjects in whom 3 or more of the following 5 variables exceeded the cut points set out by the harmonised guidelines (2): waist circumference, blood pressure, glucose, triglyceride and HDL levels; data expressed as mean ± SD or median [interquartile range] or % (95% CIs)
